# Clinical and Angiographic Predictors of No‐Reflow and Its Impact on Outcomes in ST‐Elevation Myocardial Infarction: A Retrospective Study

**DOI:** 10.1002/clc.70374

**Published:** 2026-06-15

**Authors:** Husnain Bashir, F. N. U. Berkha, Jahanzeb Malik, Abida Perveen

**Affiliations:** ^1^ Department of Medicine Ibn e Seena Hospital Kabul Afghanistan; ^2^ Department of Cardiology Abbas Institute of Medical Sciences Muzaffarabad Pakistan

**Keywords:** major adverse cardiovascular events, no‐reflow phenomenon, predictors, primary percutaneous coronary intervention, ST‐elevation myocardial infarction

## Abstract

**Objective:**

To identify clinical and angiographic predictors of the no‐reflow phenomenon and evaluate its impact on short‐ and long‐term outcomes in patients undergoing primary percutaneous coronary intervention (PCI) for ST‐elevation myocardial infarction (STEMI).

**Methods:**

This retrospective study included 2925 patients who underwent primary PCI for STEMI between January 2020 and July 2025. Patients were stratified into no‐reflow and normal flow groups based on final TIMI flow. Baseline clinical, angiographic, and procedural data were analyzed. Multivariate logistic regression identified independent predictors of no‐reflow.

**Results:**

The no‐reflow phenomenon occurred in 526 patients (18%). Independent predictors included older age, diabetes mellitus, Killip class ≥ II, high thrombus burden, pre‐PCI TIMI 0 flow, and longer symptom‐to‐door time. Use of GP IIb/IIIa inhibitors was protective. No‐reflow patients had higher in‐hospital mortality (17.3% vs. 4.5%), lower LVEF, and increased 1‐year MACE (40.5% vs. 16.4%). Kaplan–Meier curves confirmed significantly reduced MACE‐free survival (log‐rank *p* < 0.001).

**Conclusion:**

The no‐reflow phenomenon is associated with adverse outcomes and can be predicted using routine clinical and angiographic features. Early identification may enable targeted preventive strategies.

## Graphical abstract

In 2925 STEMI patients undergoing primary PCI, no‐reflow occurred in 18% and was predicted by age, diabetes, Killip class, thrombus burden, TIMI 0 flow, and delayed presentation. No reflow was associated with higher mortality, complications, and MACE. Early identification using routine parameters may enable targeted strategies to improve outcomes.

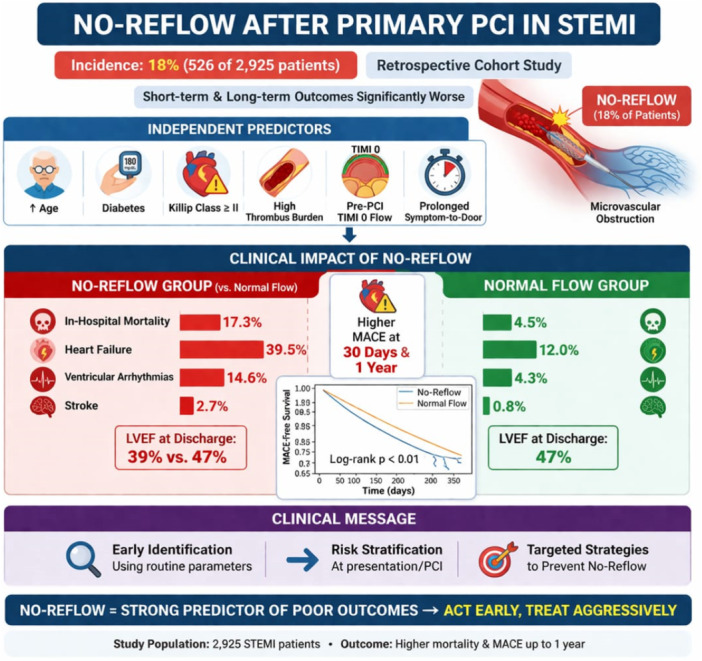

## 1 Introduction

Primary percutaneous coronary intervention (PCI) is the cornerstone of reperfusion therapy for patients presenting with ST‐elevation myocardial infarction (STEMI) and has significantly improved survival rates and myocardial salvage [1]. However, despite successful restoration of epicardial coronary flow, a subset of patients experience inadequate myocardial perfusion at the microvascular level, a phenomenon known as “no‐reflow” [2]. Angiographically defined as a thrombolysis in myocardial infarction (TIMI) flow grade < 3 in the absence of mechanical obstruction, no‐reflow occurs in approximately 5%–25% of primary PCI cases and is associated with adverse short‐ and long‐term outcomes [3, 4].

The pathophysiology of no‐reflow is complex and multifactorial, involving distal embolization of thrombotic debris, ischemia–reperfusion injury, microvascular dysfunction, and endothelial damage [5]. Several studies have investigated predictors of no‐reflow, consistently identifying factors such as advanced age, diabetes mellitus, prolonged ischemic time, high thrombus burden, and poor pre‐procedural TIMI flow as key contributors [6, 7, 8, 9]. In addition, emerging evidence suggests that inflammatory and hematologic markers may further enhance risk prediction. However, the relative importance and reproducibility of these predictors vary across different populations and clinical settings.

Clinically, the occurrence of no‐reflow has been independently associated with increased in‐hospital mortality, larger infarct size, reduced left ventricular function, and a higher incidence of heart failure and major adverse cardiovascular events (MACE) [7, 10]. Despite these well‐established associations, data from South Asian populations remain limited, and few studies have comprehensively evaluated both predictors and long‐term outcomes in large contemporary cohorts.

Therefore, the present study aims to address this knowledge gap by systematically evaluating clinical and angiographic predictors of the no‐reflow phenomenon and examining its impact on in‐hospital and long‐term outcomes in a large cohort of patients undergoing primary PCI for STEMI.

## 2 Methods

### 2.1 Study Design and Setting

This was a single‐center, retrospective cohort study conducted at the Department of Cardiology, Abbas Institute of Medical Sciences, Muzaffarabad, Pakistan. The study was approved by the Institutional Review Board of Abbas Institute of Medical Sciences (Study ID: AIMS/25/34) and adhered to the ethical principles of the Declaration of Helsinki. Due to the retrospective design and use of de‐identified data, the requirement for informed consent was waived.

### 2.2 Study Population

**Figure 1:**
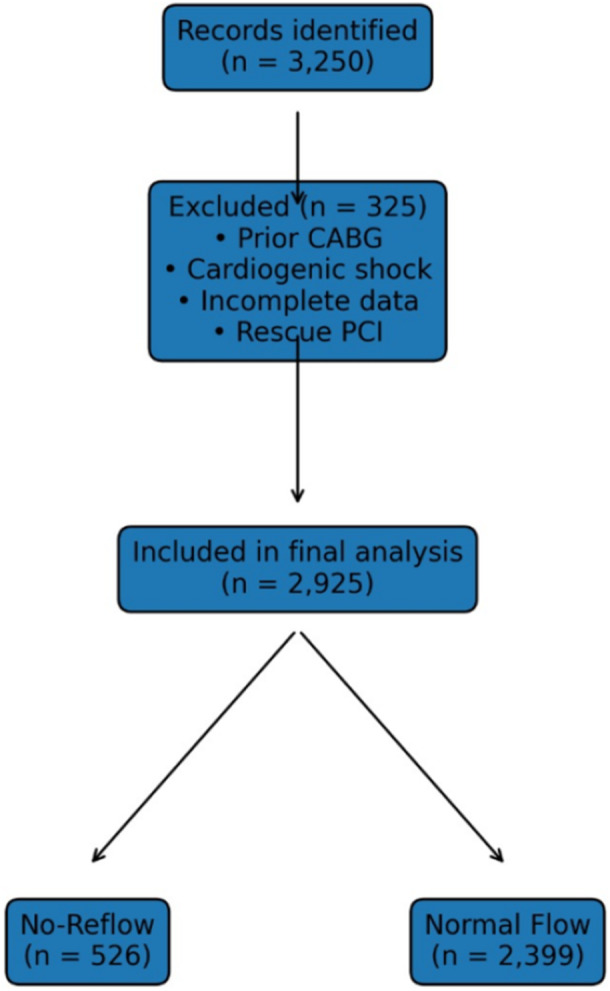
Study flow diagram illustrating the selection of patients included in the study. A total of 3250 patients presenting with ST‐elevation myocardial infarction (STEMI) and undergoing primary percutaneous coronary intervention (PCI) were screened. After exclusion of patients with prior coronary artery bypass graft surgery, cardiogenic shock, incomplete clinical or angiographic data, and those undergoing rescue PCI, 2925 patients were included in the final analysis. These were subsequently stratified into no‐reflow (*n* = 526) and normal flow (*n* = 2399) groups based on final TIMI flow grade.

We retrospectively reviewed all consecutive adult patients (≥18 years) who underwent primary PCI for STEMI between January 2020 and July 2025. STEMI was diagnosed based on typical chest pain lasting more than 30 min, ST‐segment elevation ≥ 1 mm in two or more contiguous leads on electrocardiography, and elevated cardiac biomarkers. Patients were excluded if they had prior coronary artery bypass graft (CABG) surgery, presented with cardiogenic shock, had incomplete angiographic or clinical data, or underwent rescue PCI following failed thrombolysis. The study selection process, including the number of included and excluded patients, is illustrated in a flow diagram (Figure 1).

### 2.3 Definitions and Outcome Measures

The no‐reflow phenomenon was defined as a final TIMI flow grade of 0–2 in the infarct‐related artery in the absence of mechanical obstruction (e.g., dissection, spasm, or residual thrombus), as independently assessed by two experienced interventional cardiologists. The primary outcome was the occurrence of no‐reflow. Secondary outcomes included in‐hospital complications (death, heart failure, ventricular arrhythmias, and stroke), left ventricular ejection fraction (LVEF) at discharge, and MACE at 30 days and 1 year, defined as a composite of all‐cause mortality, reinfarction, target vessel revascularization, and stroke.

High thrombus burden was assessed using the validated TIMI thrombus grading system. Thrombus grades ranged from 0 (no thrombus) to 5 (total occlusion). In cases of total occlusion (grade 5), thrombus burden was reassessed after restoration of antegrade flow. Consistent with established definitions, high thrombus burden was defined as TIMI thrombus grade ≥ 4.

### 2.4 Data Collection

Clinical, laboratory, and angiographic data were extracted from electronic medical records and catheterization laboratory databases. Recorded variables included demographic characteristics, cardiovascular risk factors (diabetes, hypertension, dyslipidemia, smoking), symptom‐to‐door and door‐to‐balloon times, Killip class on admission, baseline laboratory values (troponin, creatinine), and echocardiographic LVEF. Angiographic data included culprit vessel, lesion characteristics, thrombus grade, use of thrombus aspiration, glycoprotein IIb/IIIa inhibitors, and stent characteristics (type, length, and diameter).

### 2.5 Handling of Missing Data

Missing data were assessed for all variables. Variables with substantial missingness (>10%) were excluded from multivariable analysis. For variables with minimal missing data (<5%), complete‐case analysis was performed, as the proportion of missing values was small and unlikely to significantly impact the results.

### 2.6 Statistical Analysis

Continuous variables were expressed as mean ± standard deviation or median (interquartile range), as appropriate, and compared using the Student's *t*‐test or Mann–Whitney *U* test. Categorical variables were presented as frequencies and percentages and compared using the chi‐square or Fisher's exact test.

Univariate analyses were initially performed to identify variables associated with no‐reflow. Variables with a *p*‐value < 0.10, along with clinically relevant factors identified from prior literature, were included in the multivariable logistic regression model to determine independent predictors. To minimize the risk of overfitting, the number of variables included in the model was restricted according to the number of outcome events, maintaining an appropriate events‐per‐variable ratio.

Model performance was evaluated using measures of discrimination and calibration, including the area under the receiver operating characteristic (ROC) curve (AUC) and the Hosmer–Lemeshow goodness‐of‐fit test. Kaplan–Meier survival curves were constructed to assess MACE‐free survival at 30 days and 1 year, and comparisons were made using the log‐rank test.

A two‐sided *p*‐value < 0.05 was considered statistically significant. All statistical analyses were performed using SPSS version 26.0 (IBM Corp., Armonk, NY, USA).

## 3 Results

### 3.1 Baseline Characteristics

A total of 2925 patients with STEMI underwent primary PCI during the study period. Among them, 526 patients (18.0%) developed the no‐reflow phenomenon, whereas 2399 patients (82.0%) achieved normal epicardial coronary flow after PCI.

Patients with no‐reflow were significantly older than those with normal flow (65.4 ± 10.2 vs. 59.2 ± 11.5 years, *p* < 0.001) and were more frequently male (80.5% vs. 73.1%, *p* < 0.001). Cardiovascular risk factors were more prevalent in the no‐reflow group, including hypertension (60.1% vs. 42.6%), diabetes mellitus (55.0% vs. 33.5%), and active smoking (50.4% vs. 36.6%) (all *p* < 0.001). Additionally, patients with no‐reflow more commonly presented with Killip class ≥ II (38.3% vs. 15.7%, *p* < 0.001) and had longer ischemic time intervals, including symptom‐to‐door time (289 ± 62 vs. 236 ± 51 min, *p* < 0.001) and door‐to‐balloon time (84 ± 17 vs. 72 ± 16 min, *p* < 0.001). Baseline renal function was worse in the no‐reflow group, as reflected by higher creatinine levels (1.3 ± 0.4 vs. 1.0 ± 0.3 mg/dL, *p* < 0.001), and these patients also had lower LVEF at admission (38% ± 7% vs. 44% ± 6%, *p* < 0.001) (Table 1).

**Table 1:** Baseline clinical characteristics of patients undergoing primary percutaneous coronary intervention stratified by no‐reflow status.

Variable	No‐reflow (*n* = 526)	Normal flow (*n* = 2399)	*p* value
Sample Size	526	2399	—
Age (years), mean ± SD	65.4 ± 10.2	59.2 ± 11.5	<0.001
Male, *n* (%)	426 (80.5%)	1930 (73.1%)	<0.001
Hypertension, *n* (%)	318 (60.1%)	1125 (42.6%)	<0.001
Diabetes Mellitus, *n* (%)	291 (55.0%)	885 (33.5%)	<0.001
Smoker, *n* (%)	267 (50.4%)	967 (36.6%)	<0.001
Killip Class ≥ II, *n* (%)	203 (38.3%)	414 (15.7%)	<0.001
Symptom to Door Time (min), mean ± SD	289 ± 62	236 ± 51	<0.001
Door to Balloon Time (min), mean ± SD	84 ± 17	72 ± 16	<0.001
Creatinine (mg/dL), mean ± SD	1.3 ± 0.4	1.0 ± 0.3	<0.001
LVEF at Admission (%), mean ± SD	38 ± 7	44 ± 6	<0.001

*Note:* This table presents the baseline demographic and clinical characteristics of patients with ST‐elevation myocardial infarction (STEMI) who underwent primary PCI between January 2020 and July 2025, stratified by the presence or absence of the no‐reflow phenomenon. Continuous variables are expressed as mean ± standard deviation, and categorical variables are presented as number and percentage. The no‐reflow phenomenon was defined as a final TIMI flow grade of 0–2 in the absence of mechanical obstruction. Significant differences were observed between the groups in terms of age, cardiovascular risk factors, Killip class, ischemic time intervals, and renal function.

### 3.2 Angiographic and Procedural Characteristics

Angiographic findings demonstrated important differences between groups. The left anterior descending artery was more frequently the culprit vessel in patients with no‐reflow (51.9% vs. 44.5%, *p* = 0.002), whereas the right coronary artery was more commonly involved in the normal flow group (38.8% vs. 33.6%, *p* = 0.03). Multivessel coronary artery disease was significantly more prevalent among patients with no‐reflow (54.0% vs. 34.8%, *p* < 0.001).

A markedly higher proportion of patients in the no‐reflow group exhibited high thrombus burden (66.7% vs. 32.9%, *p* < 0.001) and pre‐procedural TIMI 0 flow (88.8% vs. 72.3%, *p* < 0.001). Procedural characteristics also differed, with more frequent use of thrombus aspiration (56.9% vs. 33.1%, *p* < 0.001) and glycoprotein IIb/IIIa inhibitors (76.2% vs. 37.6%, *p* < 0.001) in the no‐reflow group. In addition, patients with no‐reflow received longer stents (34.2 ± 5.8 vs. 28.7 ± 6.2 mm, *p* < 0.001) with smaller diameters (2.8 ± 0.4 vs. 3.0 ± 0.5 mm, *p* < 0.001) (Table 2).

**Table 2:** Angiographic and procedural characteristics of patients undergoing primary PCI stratified by no‐reflow status.

Variable	No‐reflow (*n* = 526)	Normal flow (*n* = 2399)	*p* value
Culprit vessel: LAD, *n* (%)	273 (51.9%)	1067 (44.5%)	0.002
Culprit vessel: RCA, *n* (%)	177 (33.6%)	931 (38.8%)	0.03
Culprit vessel: LCx, *n* (%)	76 (14.5%)	401 (16.7%)	0.18
Multivessel disease, *n* (%)	284 (54.0%)	835 (34.8%)	<0.001
High thrombus burden (Grade ≥ 4), *n* (%)	351 (66.7%)	790 (32.9%)	<0.001
Pre‐PCI TIMI 0 flow, *n* (%)	467 (88.8%)	1,734 (72.3%)	<0.001
Use of thrombus aspiration, *n* (%)	299 (56.9%)	795 (33.1%)	<0.001
Use of GP IIb/IIIa inhibitors, *n* (%)	401 (76.2%)	902 (37.6%)	<0.001
Stent length (mm), mean ± SD	34.2 ± 5.8	28.7 ± 6.2	<0.001
Stent diameter (mm), mean ± SD	2.8 ± 0.4	3.0 ± 0.5	<0.001

*Note:* This table presents the angiographic and procedural findings of patients with ST‐elevation myocardial infarction (STEMI) undergoing primary percutaneous coronary intervention (PCI), stratified by the occurrence of the no‐reflow phenomenon. The no‐reflow group demonstrated a higher prevalence of multivessel disease, high thrombus burden (thrombus grade ≥ 4), pre‐procedural TIMI 0 flow, and more frequent use of thrombus aspiration and glycoprotein IIb/IIIa inhibitors. Stent deployment characteristics, including length and diameter, are also compared between groups.

### 3.3 Independent Predictors of No‐Reflow

Multivariate logistic regression analysis identified several independent predictors of the no‐reflow phenomenon. Increasing age was associated with a higher risk of no‐reflow (OR 1.03 per year increase, 95% CI 1.01–1.05, *p* < 0.001). Diabetes mellitus was also a significant predictor (OR 1.52, 95% CI 1.23–1.88, *p* < 0.001), as was presentation with Killip class ≥ II (OR 2.31, 95% CI 1.83–2.92, *p* < 0.001).

**Figure 2:**
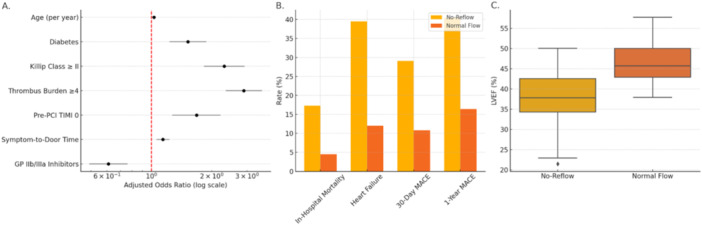
Predictors and clinical impact of the no‐reflow phenomenon in patients undergoing primary PCI. (A) Forest plot of independent predictors of no‐reflow. This panel displays a forest plot of adjusted odds ratios (ORs) with 95% confidence intervals (CIs) from the multivariate logistic regression model. Significant predictors of no‐reflow included older age, diabetes mellitus, higher Killip class (≥II), high thrombus burden (≥grade 4), pre‐procedural TIMI 0 flow, and longer symptom‐to‐door time. The use of glycoprotein IIb/IIIa inhibitors was associated with a reduced risk of no‐reflow. Odds ratios are presented on a logarithmic scale; the red dashed line indicates OR = 1. (B) Comparison of adverse outcomes between no‐reflow and normal flow groups. This bar graph compares rates of key adverse clinical outcomes—namely, in‐hospital mortality, heart failure, 30‐day major adverse cardiovascular events (MACE), and 1‐year MACE—between patients with and without the no‐reflow phenomenon. Patients in the no‐reflow group experienced significantly higher rates for all outcomes. (C) Boxplot of left ventricular ejection fraction (LVEF) at discharge by flow status. This panel illustrates the distribution of LVEF at discharge in patients with no‐reflow versus normal flow. The no‐reflow group had significantly lower LVEF, reflecting greater myocardial injury and impaired functional recovery.

Angiographic variables showed strong associations, particularly high thrombus burden (OR 2.89, 95% CI 2.35–3.56, *p* < 0.001) and pre‐PCI TIMI 0 flow (OR 1.68, 95% CI 1.27–2.21, *p* < 0.001). Prolonged symptom‐to‐door time was also independently associated with no‐reflow (OR 1.14 per 30‐min increase, 95% CI 1.06–1.23, *p* < 0.001). Conversely, the use of glycoprotein IIb/IIIa inhibitors was associated with a significantly reduced likelihood of developing no‐reflow (OR 0.61, 95% CI 0.49–0.76, *p* < 0.001) (Table 3; Figure 2A).

**Table 3:** Multivariate logistic regression analysis for predictors of no‐reflow.

Variable	Adjusted OR (95% CI)	*p* value
Age (per year increase)	1.03 (1.01–1.05)	<0.001
Diabetes mellitus	1.52 (1.23–1.88)	<0.001
Killip class ≥ II	2.31 (1.83–2.92)	<0.001
High thrombus burden (≥Grade 4)	2.89 (2.35–3.56)	<0.001
Pre‐PCI TIMI 0 flow	1.68 (1.27–2.21)	<0.001
Symptom to door time (per 30 min increase)	1.14 (1.06–1.23)	<0.001
Use of GP IIb/IIIa Inhibitors	0.61 (0.49–0.76)	<0.001

*Note:* This table summarizes the independent predictors of the no‐reflow phenomenon as determined by multivariate logistic regression analysis. Variables with a *p*‐value < 0.10 on univariate analysis were included in the final model. Higher age, presence of diabetes mellitus, Killip class ≥ II, high thrombus burden, pre‐PCI TIMI 0 flow, and longer symptom‐to‐door time were significantly associated with increased risk of no‐reflow. Conversely, the use of glycoprotein IIb/IIIa inhibitors was associated with a reduced likelihood of no‐reflow.

### 3.4 In‐Hospital and Follow‐Up Clinical Outcomes

Patients who developed the no‐reflow phenomenon experienced significantly worse in‐hospital outcomes. In‐hospital mortality was markedly higher in the no‐reflow group compared with the normal flow group (17.3% vs. 4.5%, *p* < 0.001). Similarly, rates of heart failure were substantially increased (39.5% vs. 12.0%, *p* < 0.001), along with ventricular arrhythmias (14.6% vs. 4.3%, *p* < 0.001) and stroke (2.7% vs. 0.8%, *p* = 0.001). Left ventricular systolic function at discharge was significantly impaired among patients with no‐reflow (LVEF 39 ± 6% vs. 47 ± 5%, *p* < 0.001) (Table 4; Figure 2B,C).

**Table 4:** N‐Hospital and 1‐year clinical outcomes stratified by no‐reflow status.

Outcome	No‐reflow (*n* = 526)	Normal flow (*n* = 2399)	*p* value
In‐hospital mortality, *n* (%)	91 (17.3%)	108 (4.5%)	<0.001
Heart failure (Killip class ≥ II), *n* (%)	208 (39.5%)	287 (12.0%)	<0.001
Ventricular arrhythmias, *n* (%)	77 (14.6%)	104 (4.3%)	<0.001
Stroke, *n* (%)	14 (2.7%)	19 (0.8%)	0.001
LVEF at discharge (%), mean ± SD	39 ± 6	47 ± 5	<0.001
30‐Day MACE, *n* (%)	153 (29.1%)	260 (10.8%)	<0.001
1‐Year MACE, *n* (%)	213 (40.5%)	394 (16.4%)	<0.001

*Note:* This table compares adverse clinical outcomes in patients with and without the no‐reflow phenomenon following primary PCI. Patients with no‐reflow experienced significantly higher rates of in‐hospital mortality, heart failure, ventricular arrhythmias, and stroke. At discharge, left ventricular ejection fraction (LVEF) was markedly reduced in the no‐reflow group. Rates of major adverse cardiovascular events (MACE) at both 30 days and 1 year were significantly higher in patients with no‐reflow.

During follow‐up, adverse cardiovascular events remained significantly more frequent in patients with no‐reflow. At 30 days, MACE occurred in 29.1% of patients in the no‐reflow group compared with 10.8% in the normal flow group (*p* < 0.001). This difference persisted at 1 year, with MACE rates of 40.5% versus 16.4%, respectively (*p* < 0.001).

**Figure 3:**
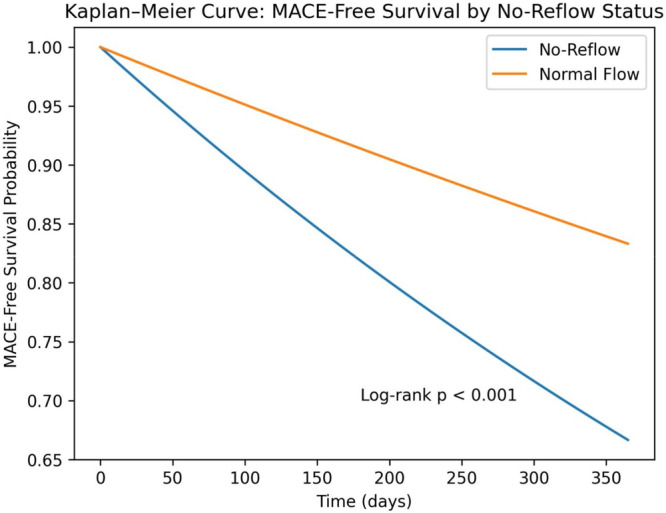
Kaplan–Meier Curve for MACE‐Free Survival in Patients With and Without the No‐Reflow Phenomenon. The figure displays Kaplan–Meier survival curves comparing the cumulative incidence of major adverse cardiovascular event (MACE)‐free survival between patients who experienced the no‐reflow phenomenon and those with normal coronary flow following primary percutaneous coronary intervention (PCI) for ST‐elevation myocardial infarction (STEMI). The follow‐up period has been revised to 1 year (365 days) to align with the reported outcomes in Table 4. Patients in the no‐reflow group demonstrated significantly lower MACE‐free survival compared to the normal flow group (log‐rank *p* < 0.001), indicating a poorer short‐ to mid‐term prognosis associated with no‐reflow.

Kaplan–Meier survival analysis demonstrated significantly lower MACE‐free survival in the no‐reflow group throughout the 1‐year follow‐up period (log‐rank *p* < 0.001), confirming the substantial adverse prognostic impact of the no‐reflow phenomenon (Figure 3).

## 4 Discussion

In this retrospective cohort of 2925 patients undergoing primary PCI for STEMI, we identified several independent predictors of the no‐reflow phenomenon, including older age, diabetes mellitus, higher Killip class (≥II), high thrombus burden, pre‐PCI TIMI 0 flow, and prolonged symptom‐to‐door time. Importantly, no‐reflow was strongly associated with adverse clinical outcomes, including increased in‐hospital mortality, higher rates of heart failure, ventricular arrhythmias, stroke, reduced LVEF, and significantly elevated 30‐day and 1‐year MACE, with persistently lower MACE‐free survival (Figures 2 and 3).

These findings are consistent with prior literature demonstrating that impaired baseline coronary flow and high thrombus burden are key determinants of no‐reflow [8]. Large STEMI cohorts have similarly identified advanced age, diabetes, delayed reperfusion, and thrombus burden as major contributors [9, 10]. Observational data further support the association between no‐reflow and worse clinical outcomes, including increased mortality and heart failure [11]. However, rather than reiterating these associations, our study emphasizes their practical applicability in routine clinical settings.

A key strength of this study is the large sample size and its focus on a South Asian population, where data on no‐reflow remain limited. By demonstrating that routinely available clinical and angiographic parameters can effectively predict no‐reflow, our findings provide a pragmatic approach to early risk stratification. This has important clinical implications, as identifying high‐risk patients at the time of presentation or during PCI may allow for the implementation of targeted preventive strategies, including optimization of reperfusion timing and tailored antithrombotic therapy.

The protective association observed with glycoprotein IIb/IIIa inhibitors aligns with previous interventional studies suggesting that these agents may reduce microvascular obstruction in patients with high thrombus burden [9]. Although this finding may be influenced by confounding factors, it supports a selective, risk‐based approach to adjunctive pharmacotherapy during PCI.

Clinically, the occurrence of no‐reflow identifies a subgroup with markedly worse prognosis. Prior studies have consistently shown that no‐reflow is associated with larger infarct size, impaired ventricular function, adverse remodeling, and increased short‐ and long‐term mortality [12, 13]. Long‐term registry data further indicate that these adverse effects may persist even in cases of transient microvascular dysfunction [14]. Our findings reinforce these observations while demonstrating their relevance in a contemporary cohort.

Overall, this study highlights the continued clinical importance of the no‐reflow phenomenon and underscores the value of early identification using simple, readily available parameters. Future research should focus on integrating clinical, imaging, and biomarker‐based approaches to refine risk prediction and evaluate targeted interventions aimed at reducing no‐reflow and improving outcomes in STEMI patients undergoing primary PCI.

In STEMI patients undergoing primary PCI, pre‐procedural platelet‐derived growth factor‐BB (PDGF‐BB) levels were significantly higher in those who developed no‐reflow and remained an independent predictor in multivariate analysis. A cutoff value of 89.99 pg/mL predicted no‐reflow with moderate accuracy (AUC = 0.688), showing high specificity (87.2%) but modest sensitivity (51.5%) [15].

Among 208 STEMI patients undergoing primary PCI, no‐reflow occurred in 26.4%. Patients with no‐reflow had significantly higher dNLR, CRP, glucose, creatinine, and troponin levels, along with lower LVEF. On multivariate analysis, dNLR, diabetes mellitus, CRP, and LVEF were identified as independent predictors of no‐reflow [16].

A study showed that among 1931 STEMI patients undergoing primary PCI, the no‐reflow phenomenon occurred in 14.1% and in‐hospital mortality in 2.5%, with admission EASIX independently predicting both outcomes and demonstrating good discriminative ability (AUC 0.706 for no‐reflow and 0.810 for mortality) [17].

## 5 Limitations

Our study has several limitations. First, its retrospective, single‑center design could introduce selection bias and limit external validity. Second, we lacked data on inflammatory biomarkers and serologic indices such as CRP, fibrinogen‐to‐albumin ratio (FAR), CRP/albumin ratio (CAR), or atherogenic index of plasma (AIP), which have been shown to predict no‑reflow and long‑term outcomes in other cohorts [11, 18]. Biomarkers reflecting endothelial dysfunction or inflammation may provide additional predictive value if available.

Third, we did not assess intracoronary imaging parameters (e.g., plaque morphology, microvascular obstruction by contrast echo or MRI) or evaluate procedural subtleties like distal embolization events or presence of collateral circulation, which were identified in other studies as relevant predictors [19]. Fourth, our study did not evaluate the impact of pre‑PCI medical therapies such as pre‑hospital ACE inhibitor or ARB use, which some studies suggest may reduce no‑reflow risk [13].

Fifth, although we observed strong associations, the use of GP IIb/IIIa inhibitors and thrombus aspiration may be confounded by indication bias: these agents were more likely administered to patients with high‑thrombus burden, so their “protective” effect may reflect selection rather than causal benefit. Prospective or randomized data would be needed to clarify this.

Sixth, 5‑year outcomes were collected via medical records and follow‑up visits; however, some events (e.g., rehospitalization elsewhere) may have been missed, leading to under‑ascertainment of MACE.

Finally, while our study included a large sample overall, the number of events in certain subgroups (e.g., stroke, arrhythmias) was relatively small, limiting power to evaluate subgroup interactions or more granular predictors.

## 6 Clinical Implications and Future Directions

These findings suggest that attention to clinical and angiographic features such as age, diabetes status, Killip class, thrombus burden, and initial TIMI flow can help identify patients at high risk of no‑reflow. In such cases, anticipatory strategies may include aggressive antithrombotic therapy, selective use of GP‑IIb/IIIa inhibitors, deferred stenting, or adjunctive pharmacologic agents (e.g., intracoronary vasodilators).

Future prospective, multi‑center studies should incorporate biomarker data (CAR, FAR, AIP), intravascular imaging, and measure long‐term remodeling outcomes. Randomized trials evaluating targeted preventive strategies in high‐risk subsets are warranted. Additionally, validation of predictive models in diverse ethnic and geographic populations (such as South Asian cohorts) would improve generalizability.

## 7 Conclusion

In conclusion, no‑reflow following primary PCI for STEMI remains a significant challenge and portends worse both acute and long‑term outcomes. Our study confirms that simple clinical and angiographic variables available at presentation can predict no‑reflow risk. Identifying and proactively managing high‑risk patients may help mitigate microvascular injury and improve prognosis.

## Author Contributions

**Husnain Bashir:** writing and supervision. **F.N.U. Berkha:** conceptualization, methodology, writing. **Jahanzeb Malik:** writing, validation, software, investigation. **Abida Perveen:** formal analysis, data correction.

## Funding

The authors have nothing to report.

## Conflicts of Interest

The authors declare no conflicts of interest.

## Data Availability

The data that support the findings of this study are available from the corresponding author upon reasonable request. Data is available upon request.
